# Full-Thickness Macular Hole Due to Choroidal Neovascularization in the Setting of Pathologic Myopia

**DOI:** 10.1177/24741264221104592

**Published:** 2022-08-08

**Authors:** Austin Pereira, Brian G. Ballios, David Sarraf, Peng Yan

**Affiliations:** 1 Department of Ophthalmology and Vision Sciences, University of Toronto, Toronto, ON, Canada; 2 Kensington Vision and Research Center, University of Toronto, Toronto, ON, Canada; 3 Department of Ophthalmology, University Health Network, Toronto, ON, Canada; 4 Stein Eye Institute, University of California, Los Angeles, CA, USA

**Keywords:** anti-VEGF, CNV, dehiscence, macular hole, myopia, neovascularization

## Abstract

**Purpose:** To present a case of myopic choroidal neovascularization (CNV) leading to a full-thickness macular hole (MH) in a patient with macular schisis. **Methods:** A single case was evaluated. **Results:** A 65-year-old woman presented with myopic staphyloma and foveoschisis in both eyes. One month after the baseline presentation for myopic macular schisis, the patient presented with a paracentral scotoma in the left eye. Examination showed a submacular hemorrhage in the left eye. Optical coherence tomography of the left eye showed subretinal fluid and subretinal hyperreflective material in the fovea, suggestive of exudative myopia, and a small full-thickness MH (diameter 86 µm). After anti-vascular endothelial growth factor injections, the CNV showed interval improvement; however, a larger full-thickness MH (diameter 287 µm) developed in the left eye. **Conclusions:** A full-thickness MH developed secondary to CNV, leading to foveal dehiscence in an eye with baseline macular schisis.

## Introduction

Macular holes (MHs) are breaks in the neurosensory retina primarily caused by anteroposterior traction of the posterior hyaloid onto the inner retinal interface. This ultimately leads to vitreoretinal traction and foveal dehiscence. Specialized central Müller cells, which span the entirety of the neurosensory retina, play a crucial role in forming the floor of the fovea and stabilizing this region by adhering to neighboring foveal cone cells. In cases of pathologic myopia, foveal dehiscence can occur as a result of disruption and stress on central Müller cells, thereby predisposing patients at baseline to MH development. Biomarkers such as foveal hyperreflective stress lines on optical coherence tomography (OCT) imaging indicate this pathophysiologic mechanism.^1^ To this end, patients with myopic macular schisis caused by pathologic myopia are at high risk for progressing to full-thickness MHs.

We describe a patient who presented with a full-thickness MH and foveal dehiscence caused by mechanical elevation of an exudative myopic choroidal neovascularization (CNV) membrane developing in the setting of myopic macular schisis. To our knowledge, this is the first report of such a case.

## Case Report

A 65-year-old woman was referred to a tertiary retina clinic for assessment of myopic degeneration in both eyes. The patient reported progressively worsening blurred central vision in both eyes occurring over the past 8 years. Her medical history was significant for hypercholesterolemia and diet-controlled type 2 diabetes mellitus. There was no ocular history other than spectacle correction of high myopia (−9.25 D in right eye; −8.75 D in left eye). No relevant family history was noted.

**Figure 1.**
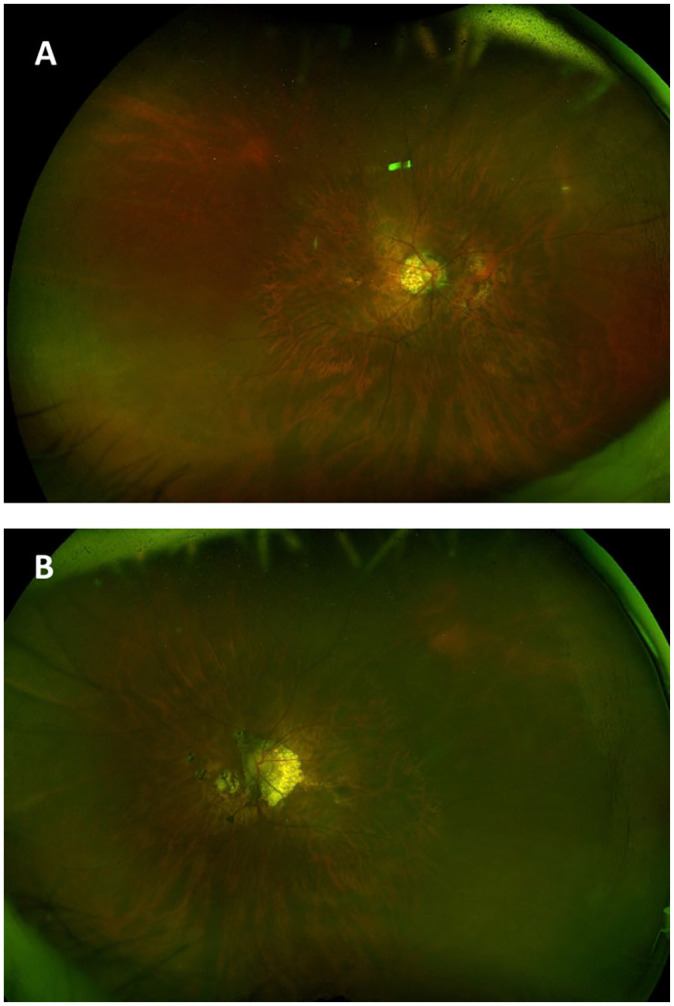
Widefield fundus imaging of the (A) right eye and (B) left eye shows a myopic-appearing fundus with tilted discs and peripapillary atrophy extending into the nasal macula in both eyes. The vessels in both eyes appear normal. The macula shows attenuated pigmentation consistent with posterior staphyloma as well as discrete foci of chorioretinal atrophy with some pigment clumping nasal to the disc in both eyes, suggestive of myopic degeneration.

**Figure 2.**
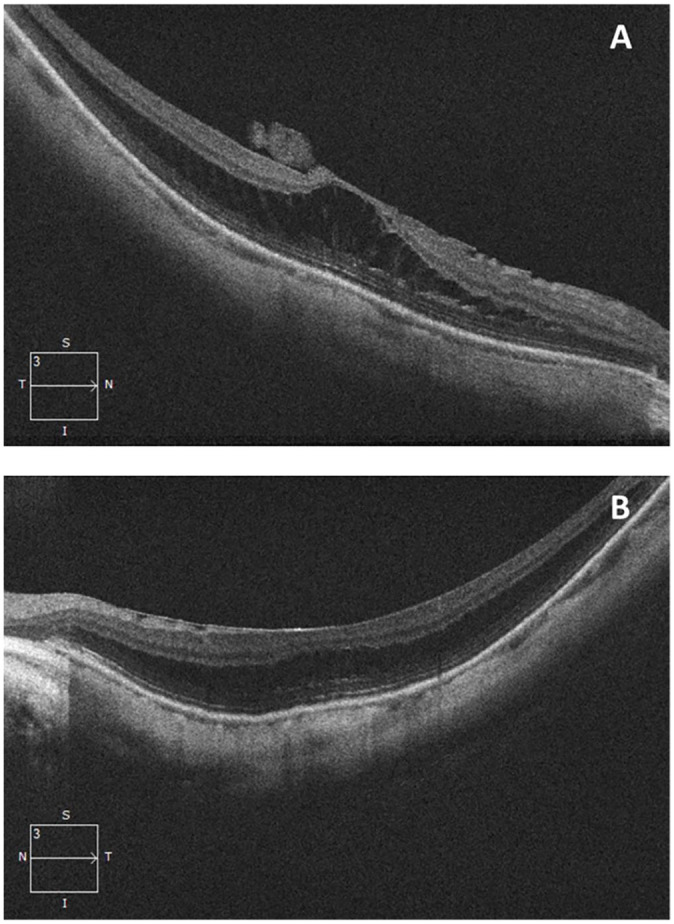
Spectral-domain optical coherence tomography line scans through the macula at initial presentation, confirming the presence of posterior staphyloma and myopic foveoschisis as well as epiretinal membranes in the (A) right eye and (B) left eye. No vitreomacular traction was present in the left eye.

On examination, the best-corrected distance visual acuity (BCVA) was 20/70 OD and 20/50 OS with no improvement on pinhole occlusion. The intraocular pressure (IOP) was 13 mm Hg and 14 mm Hg, respectively. The pupil examination was unremarkable. A slitlamp examination of the anterior segment was within normal limits with a clear crystalline lens in both eyes. Fundus examination showed bilateral macular posterior staphyloma with tilted discs and peripapillary atrophy in both eyes (Figure 1, A and B). The retina was flat bilaterally with no breaks noted on a full peripheral examination. Spectral-domain OCT of the macula confirmed the presence of a posterior staphyloma, myopic foveoschisis, and a mild epiretinal membrane (ERM) in both eyes (Figure 2, A and B). No vitreomacular traction was present in the left eye. The patient elected for observation with close follow-up.

**Figure 3.**
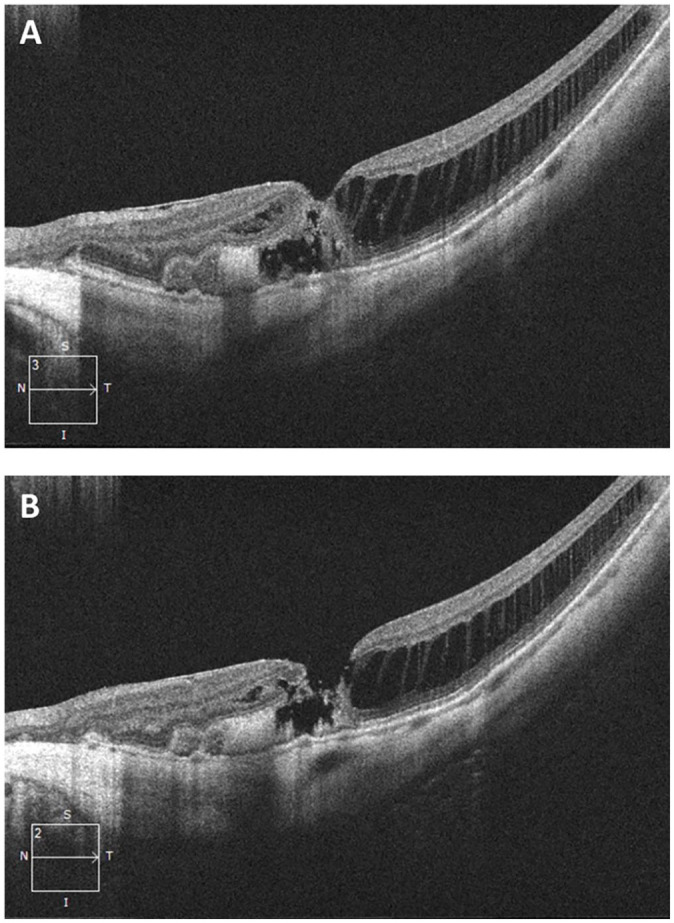
Spectral-domain optical coherence tomography line scans through the macula in the left eye 1 month after initial presentation show (A) evidence of subfoveal fluid extending into the nasal parafovea as well as a shallow underlying pigment epithelial detachment with subretinal hyperreflective material corresponding to the submacular hemorrhage. These findings are characteristic of myopic choroidal neovascularization. (B) This scan shows a stage 2 full-thickness macular hole with underlying subretinal hyperreflective material.

One month after initial presentation, the patient presented with a 2-week complaint of paracentral scotoma in the left eye. The BCVA was reduced to 20/150 OS. Subretinal fluid and hemorrhage were identified on examination, and fluid as well as subretinal hyperreflective material (SHRM) was seen on OCT imaging of the left eye (Figure 3A). A small full-thickness MH (diameter 86 µm) was also noted on OCT (Figure 3B). The presence of a partial or full-thickness MH was not present on examination or imaging at the previous visit and was likely secondary to the exudative process. The patient received intravitreal aflibercept treatment for the management of the myopic CNV.

**Figure 4.**
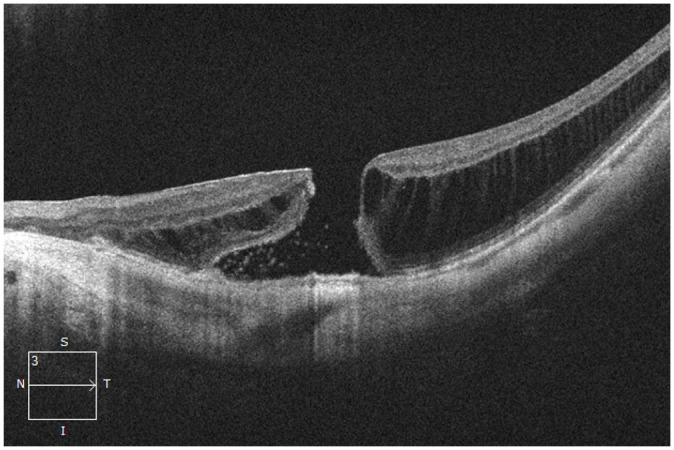
Spectral-domain optical coherence tomography of the macula in the left eye 1 month after 2 intravitreal aflibercept injections shows resolution of the subretinal hyperreflective material and a larger stage 3 full-thickness macular hole with progressed foveal dehiscence.

**Figure 5.**
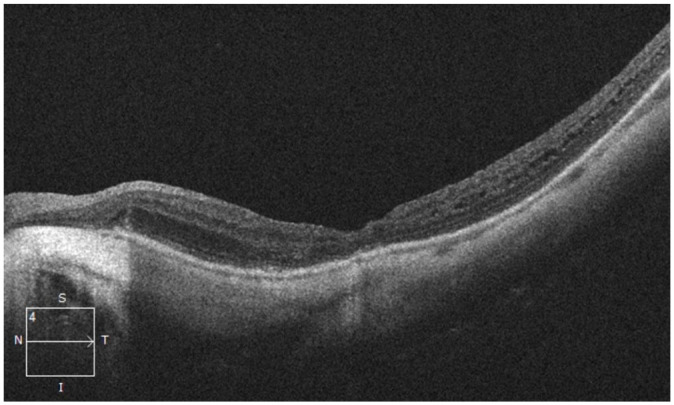
Spectral-domain optical coherence tomography macula of the left eye 1 month after the second procedure (pars plana vitrectomy with internal limiting membrane autologous transplantation) shows successful closure of the full-thickness macular hole.

After 2 intravitreal injections of aflibercept over 2 months, the patient presented with 1 week of worsening of metamorphopsia in the left eye. The BCVA was 20/200 OS, and OCT of the macula showed resolving SHRM in that eye. However, interval enlargement to a medium full-thickness MH (diameter 287 µm) and worsening foveal dehiscence were now seen (Figure 4). The intravitreal aflibercept injections were discontinued, and the patient had a pars plana vitrectomy (PPV) with 20% sulfur hexafluoride (SF_6_) gas tamponade and internal limiting membrane (ILM) peeling. However, the MH remained open after the initial procedure. A follow-up PPV with 15% perfluoropropane (C_3_F_8_) and an ILM autologous transplantation were performed 2 months after the initial surgery. The MH was closed by 1 week postoperatively. At 4 weeks, the BCVA was 20/150 and there was a visually significant cataract (Figure 5). By 3 months postoperatively, the MH had not reopened and the CNV had not recurred.

## Conclusions

To our knowledge, we are the first to describe a full-thickness MH secondary to myopic CNV in the setting of macular schisis caused by pathologic myopia. There is a wide range of etiologies for MHs, including age, trauma, vitreomacular traction (VMT), and proliferative vitreoretinopathy after PPV. Pathologic myopia is also a well-known cause of MH development because it increases the axial length, causing tangential force on the retina and ultimately inducing mechanical stress and retinoschisis.

Our patient initially presented with a notable posterior staphyloma and myopic retinoschisis, which was confirmed on OCT. Therefore, her baseline risk for developing an MH was approximately 8.4% based on population studies.^2^ When the patient presented 1 month later with decreased BCVA in the left eye (from 20/50 to 20/150), a small MH was seen over new myopic CNV (see Figure 3). We hypothesize that the anteroposterior mechanical elevation of subretinal fluid resulting from the exudative myopic CNV led to dehiscence of the already weakened neurosensory retina at the fovea. Stress placed on the central Müller cells caused by the submacular hemorrhage ultimately led to further foveal dehiscence and MH development.^1^ This force is analogous, albeit in an opposite direction, to the pathophysiology of MH development seen in VMT, in which anteroposterior tractional forces pull the retinal layers apart. With respect to other potential risk factors, aside from a mild ERM and foveoschisis at baseline, no VMT was seen on initial presentation or follow-up. In addition, the patient denied a history of ocular trauma, and no intravitreal injections were administered between the initial presentation and the first identification of the MH.

In our patient, the progression to a medium MH (diameter 86 µm to 287 µm) after 2 aflibercept injections raises the question of the effect of intravitreal antivascular endothelial growth factor (anti-VEGF) treatment on MH development and progression, as previously reported.^3–6^ Only 4 cases exist in the literature, and these describe the development of full-thickness MHs after anti-VEGF treatment for myopic CNV. Table 1 compares the demographic, clinical examination, and surgical outcome information for our patient and the 4 similar cases.^3–6^ The key difference in our case is that a small MH developed before rather than after the initiation of intravitreal anti-VEGF therapy. We believe the MH was the result of the mechanical elevation of exudative CNV.

**Table 1.** Comparison of Current Case and 4 Other Published Cases.

Study	Age (Y)	Sex	BCVA	Treatment	Management	MH Closed	Rx (D)	PVD
Current^a^	65	F	20/200	2 doses of intravitreal aflibercept	PPV with autologous membrane transplant + 15% C_3_F_8_	Yes	−8.75	No
Chung and Koh^3^	58	F	CF OD	Photodynamic therapy + 2 doses of intravitreal bevacizumab	PPV with membrane peel + silicone oil	Yes	−17.00	No
Miura et al^4^	55	F	20/200	2 doses of intravitreal bevacizumab	Observation	Yes	−18.00	No
Otsuka et al^5^	67	M	20/200	1 dose of intravitreal ranibizumab	PPV with membrane peel + 20% SF_6_ gas	Yes	N/A	No
Sun et al^6^	60	F	CF OS	1 dose of intravitreal conbercept	PPV with membrane peel + silicone oil	No	N/A^b^	No

Abbreviations: BCVA, best-corrected visual acuity; C_3_F_8_, perfluoropropane; CF, counting fingers; MH, macular hole; N/A, not available; PPV, pars plana vitrectomy; Rx, refraction; SF_6_, sulfur hexafluoride.

a Patient had an MH before initiation of antivascular endothelial growth factor injection.

b Axial length was 30.43 mm.

Miura et al^4^ postulated that the preexisting VMT in their patient might have induced the MH after the anti-VEGF treatment led to recession of the CNV membrane. In contrast, Lee and Kim^7^ postulated that vitreous incarceration might be induced by intravitreal administration, ultimately enhancing VMT and increasing the chance of MH development. However, our patient did not have evidence of VMT on OCT at any point during presentation or treatment. In their case, Chung and Koh^3^ used intravitreal bevacizumab, whereas Otsuka et al^5^ used ranibizumab injections. This suggests that the risk of MH development after intravitreal injection might be independent of the medication itself. We hypothesize that in our case the presence of an ERM, which leads to tangential parafoveal traction, combined with CNV membrane recession led to interval MH enlargement after anti-VEGF administration.

Surgical planning for a medium MH is difficult in a patient with pathologic myopia. Our decision on surgical intervention was guided by the success seen with hole closure after surgery in 3 previous cases of myopic CNV and a full-thickness MH after anti-VEGF injections.^3,5,6^ The patient in the Miura et al^4^ report elected for observation; the full-thickness MH spontaneously closed by 6 months after initial presentation. Two of the remaining 3 cases had successful MH closure with PPV, 1 with silicone oil^3^ and 1 with 20% SF_6_ gas tamponade.^5^ The case by reported Sun et al^6^ had persistent MH after PPV with silicone oil. All 3 patients had concomitant membrane peeling during surgery.

A 2017 review by Abbey et al^8^ showed the importance of ERM peeling to minimize MH recurrence, regardless of whether the ILM is peeled before PPV. We elected to schedule our patient for PPV with ILM peeling and 20% SF_6_ gas to induce MH closure and minimize the chance of recurrence. Unfortunately, the MH remained open after the initial surgery; however, after 15% C_3_F_8_ and ILM autologous transplantation, good neurosensory retina closure was obtained.^9^ Based on our experience with myopic MHs and this case, we recommend performing an initial PPV with ILM flap surgery to increase the likelihood of hole closure for full-thickness MHs induced by CNV and worsened by anti-VEGF injections.^10^

In conclusion, we report a full-thickness MH secondary to the development of exudative CNV in the setting of pathologic myopia. We hypothesize that the anteroposterior mechanical elevation of the subretinal space led to dehiscence of the neurosensory retina in the fovea in the context of preexisting myopic foveoschisis and posterior staphyloma. Our case shows the progression of the MH after intravitreal anti-VEGF therapy for myopic CNV, as has been described in the literature.

A review of the literature confirmed that the standard management of a myopic full-thickness MH that has worsened after anti-VEGF injections is PPV with membrane peeling, although the outcomes of MH induced by intravitreal anti-VEGF have been mixed in the literature. ILM flap or autologous ILM transplant surgeries might add to the success of MH closure in these cases. Although exceedingly rare, the etiology of MH formation should be extended to include CNV, especially in patients with preexisting pathologic myopia and myopic foveoschisis.
